# Biologically synthesized iron nanoparticles (FeNPs) from *Phoenix dactylifera* have anti-bacterial activities

**DOI:** 10.1038/s41598-021-01374-4

**Published:** 2021-11-11

**Authors:** Faryal Batool, Muhammad Shahid Iqbal, Salah-Ud-Din Khan, Javed Khan, Bilal Ahmed, Muhammad Imran Qadir

**Affiliations:** 1 Institute of Molecular Biology and Biotechnology, Bahauddin Zakariya University, Multan, Pakistan; 2 Department of Clinical Pharmacy, College of Pharmacy, Prince Sattam Bin Abdulaziz University, Al-Kharj, 11942 Saudi Arabia; 3 Department of Biochemistry, College of Medicine, Imam Mohammad Ibn Saud Islamic University (IMSIU), Riyadh, 11432 Saudi Arabia; 4 Department of Public Health, College of Health Sciences, Saudi Electronic University, Riyadh, 11673 Saudi Arabia; 5 Department of Clinical Pharmacology, School of Pharmacy, Nanjing Medical University, Jiangsu Province, Nanjing, People’s Republic of China

**Keywords:** Bioinorganic chemistry, Nanobiotechnology

## Abstract

Nanotechnology is a vast field of science with the most vibrant and conspicuous applications. The green synthesis approach is cost-effective, eco-friendly, and produces the most stable metal-based nanoparticles without the use of toxic chemicals. This study presents the green synthesis of iron nanoparticles (FeNPs). For biosynthesis of FeNPs, *Phoenix dactylifera* extract was used as a reducing agent and iron sulfate heptahydrate (FeSO_4_·7H_2_O) was used as a substrate. FeNPs were characterized by different techniques including UV–Visible spectroscopy, Fourier transform infrared spectroscopy (FTIR), and nano zeta-sizer analysis. The antimicrobial activity of FeNPs synthesized by using an aqueous extract of *Phoenix dactylifera* was evaluated against *Escherichia coli*, *Bacillus subtilis*, *Micrococcus leutus,* and *Klebsiella pneumoniae*. A notable color change from yellow to black confirmed the synthesis of FeNPs. The sharp peak at 450 nm UV–Visible spectroscopy confirmed the synthesis of FeNPs. FTIR showed the presence of O–H and C=C stretching due to the presence of phenol and alkene functional groups. The average size of FeNPs was 6092 d.nm. The results of antimicrobial activity showed that FeNPs exhibit different potential against different bacterial strains with a maximum 25 ± 0.360 zone of inhibition against *Escherichia coli.* Thus, green synthesized FeNPs could be used as potential antimicrobial agents.

## Introduction

Nanotechnology is a revolutionary approach that involves the management of atoms and molecules at the nanoscale^1^. Nanotechnology has emerged as an exciting field of research in modern sciences and provides different types of products including nanoparticles, nanorods, or nanotubes with different dimensions. All these nano-sized products have different specific roles. They may vary according to their size and shape, chemical nature, and crystalline, amorphous, and solid-state of occurrence^2^. Nanoparticles have different properties in contrast to their bulk materials because of their occurrence in nanoscale^3^. Metallic nanoparticles are widely used in several fields including the textile industry, food industry, agriculture, health sector, and cosmetics. They also exhibit high surface area due to their extremely small size. The importance of these nanoparticles lies in the influence of their size on the physiochemical properties of any substance^4,5^.

Iron nanoparticles (FeNPs) are the tiniest particle of iron metal with a large surface area and high reactivity. They are non-toxic. FeNPs have excellent dimensional stability and also possess high thermal and electrical conductivity, high surface area, and are highly magnetic. FeNPs can oxidize immediately when exposed to water or air and produces free Fe ions. There are numerous applications of FeNPs but the most promising one includes their role in drug delivery.

There are several conventional approaches to synthesize FeNPs. Such conventional approaches like chemical and physical methods involve toxic and expensive chemicals and more use of energy. To reduce the use of chemicals and energy, the biological synthesis approach proves to be compatible, less expensive, less time-consuming, stable, and eco-friendly. Biological synthesis involves fungi, bacteria, viruses, and plants as reducing agents. Among all these sources, the plant-based green synthesis approach gains more attention due to the easy handling of plants^6–8^. Green synthesis of nanoparticles using plant biomaterials include different parts of the plant-like stem, leaves, roots, fruits, and seeds. Synthesizing nanoparticles by plant materials is the most familiar, simple, and cost-effective approach^3,9^. Plants produce more stable nanoparticles as compared to microorganisms. Plants are naturally composed of several organic reducing compounds, enhancing the ability of plants to synthesize nanoparticles^3,10^. A symbiotic relationship is generated between nanotechnology and plant sciences due to the usage of plant phytochemicals in the synthesis of nanoparticles. This interrelation between plant science and nanotechnology is termed “green nanotechnology”^11^.

So far, several types of plants have been used to synthesized FeNPs*, Phoenix dactylifera* plant has gained huge medicinal importance because of its versatile phytochemical composition including flavonoids, phenolics, carotenoids, sterols, procyanidins, and anthocyanins. These components are accountable for different pharmacological activities like anti-inflammatory, anti-asthmatic, anti-diabetic, antibacterial, aphrodisiac, hepatoprotective, and nephroprotective activities^12–15^. It has been recognized that leaves of *Phoenix dactylifera* showed prominent antibacterial results as compared to the seed and fruit of the plant^16^. Therefore, the objective of this study was to synthesize FeNPs by green synthesis method using *Phoenix dactylifera* leaf extract, their characterization, and evaluation of the antimicrobial activity.

## Results

**Figure 1:**
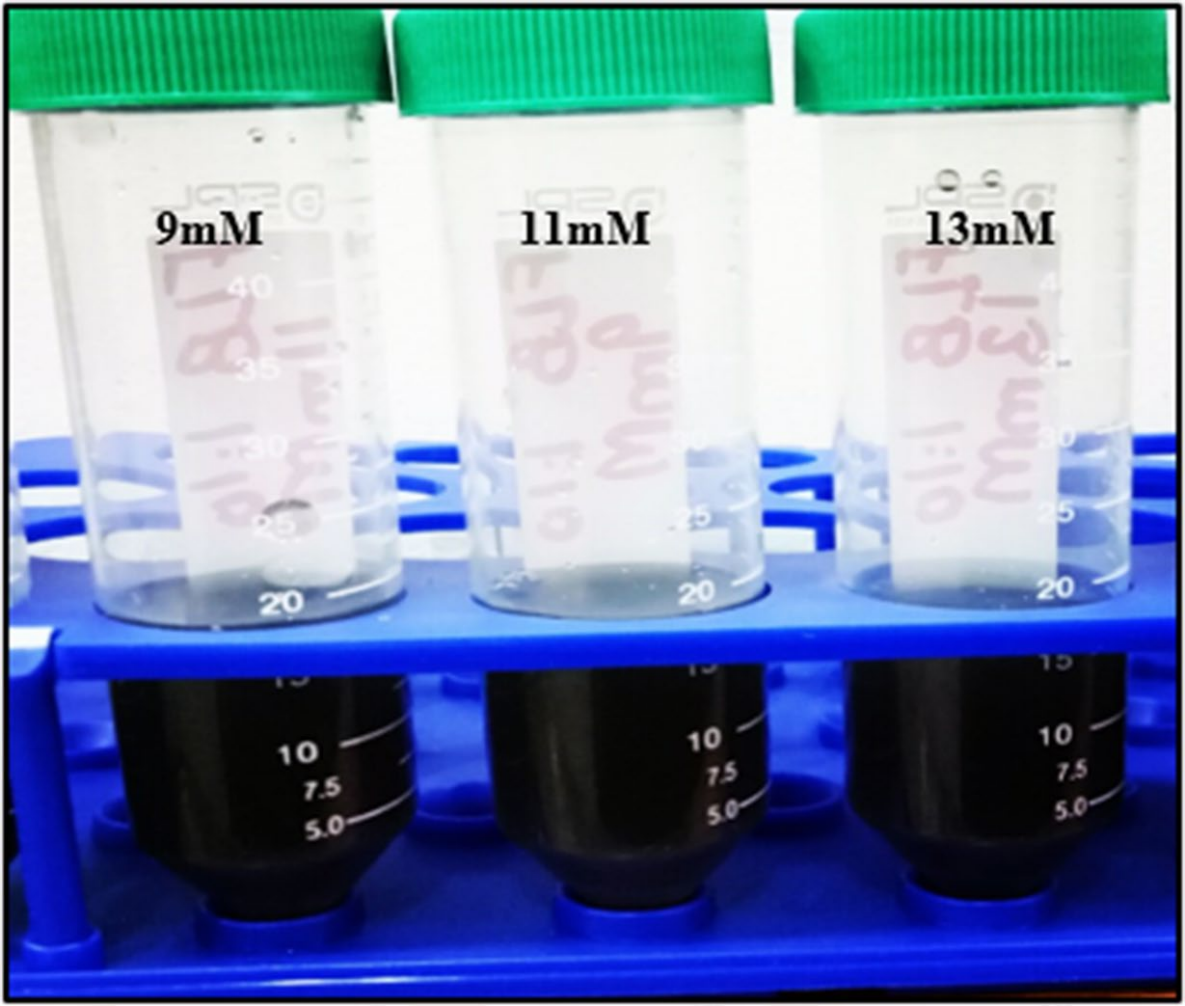
Iron Nanoparticles synthesized by *Phoenix dactylifera* leaves extracts.

FeNPs were successfully synthesized using *Phoenix dactylifera* extract by the green method. The color of the reaction mixture containing 1:10 of extract and FeSO_4_·7H_2_O changed from yellow to black after overnight shaking at 37 °C as shown in Fig. 1. The color change indicated that phytoconstituents of *Phoenix dactylifera* caused the reduction of Fe into FeNPs. This initial reduction creates a nucleation center, which leads to the accumulation of more metal ions while also incorporating the nucleation site next to it. NPs are formed as a result which becomes entrapped with biological molecules of the plant for better stability and improved morphology.

**Figure 2:**
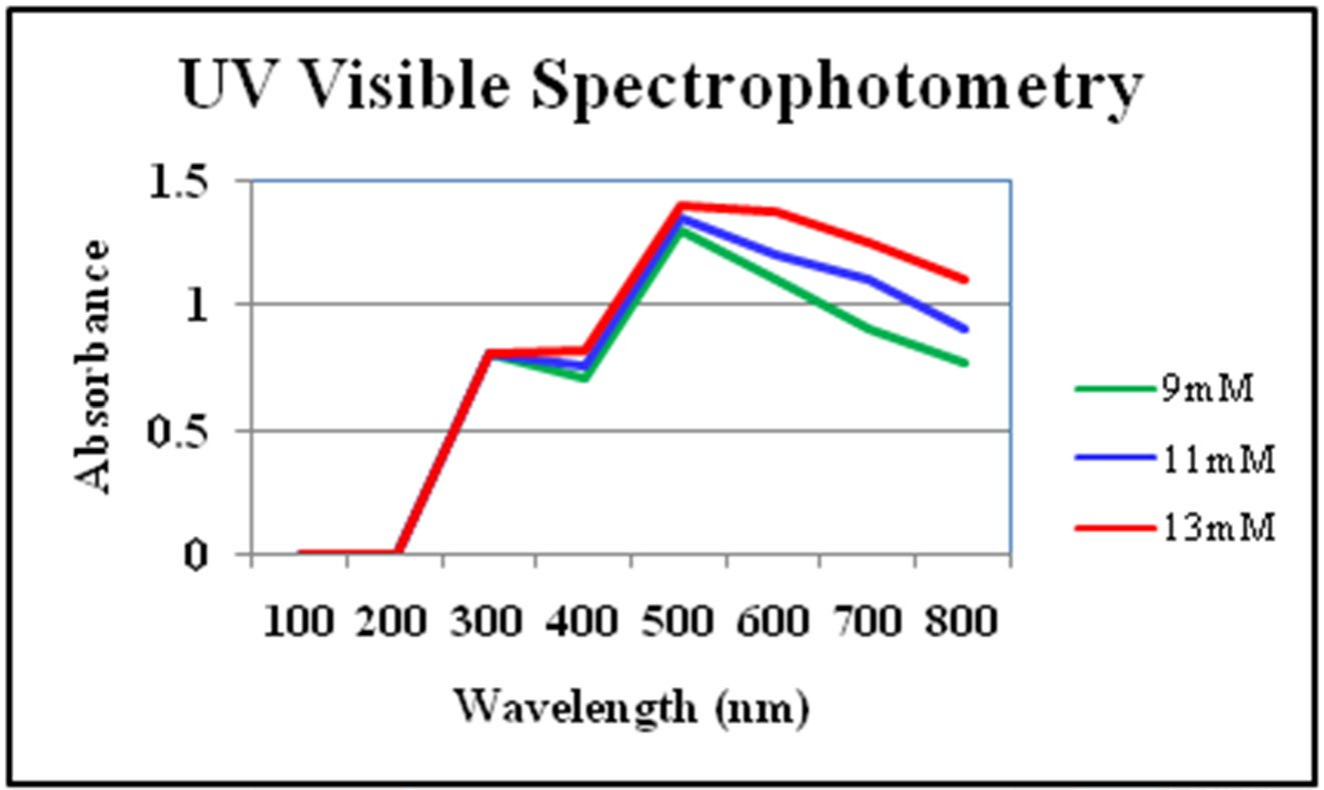
UV absorbance spectrum for FeNPs synthesized by *Phoenix dactylifera* leaves extract.

The synthesis of FeNPs was confirmed by UV–Visible analysis. The wavelength range was set at 100–800 nm. Sharp peaks were observed at 450 nm which is characteristic of FeNPs. Different absorbance peaks were observed for FeNPs synthesized from different salt concentrations. Absorbance peaks keep on increasing as the concentration of salt increases from 9 to 13 mM as shown in Fig. 2.

**Figure 3:**
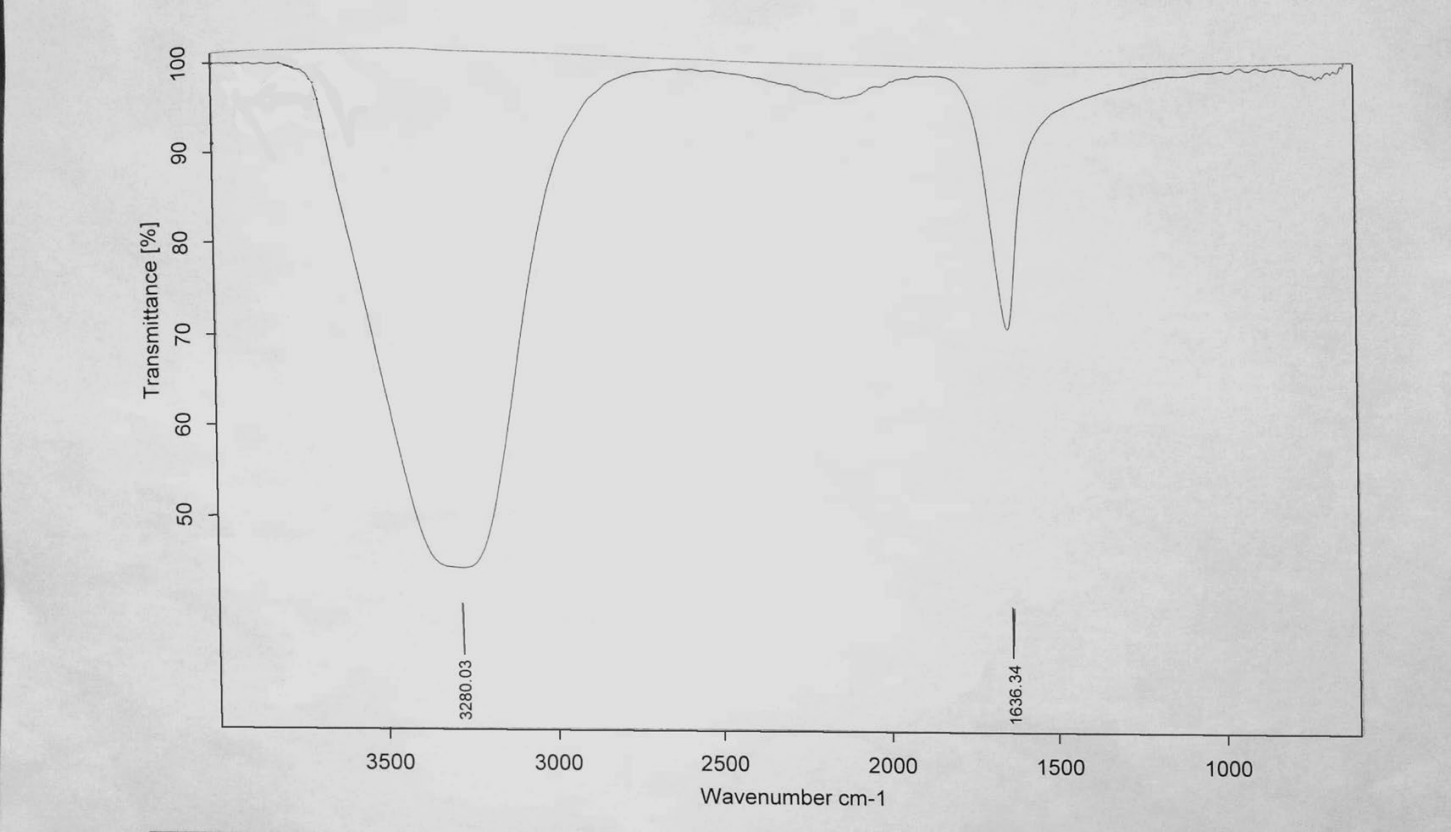
FTIR Spectrum for FeNPs synthesized by *Phoenix dactylifera* leaves extract.

FTIR spectroscopy was used to identify the presence of functional groups responsible for the synthesis of FeNPs from an aqueous extract of *Phoenix dactylifera*^10^. Figure 3 represents the absorbance bands of FeNPs in the range of wave region between 1000 and 3500 cm^-1^. Characteristics peaks were observed at 1636.34 and 3282.19 cm^-1^. The band at 3282.19 cm^-1^ represented the O–H stretching due to the presence of phenol or alcohol as a functional group and those 1636.34 cm^-1^ confirmed the C=C stretching due to the alkene functional group.

**Figure 4:**
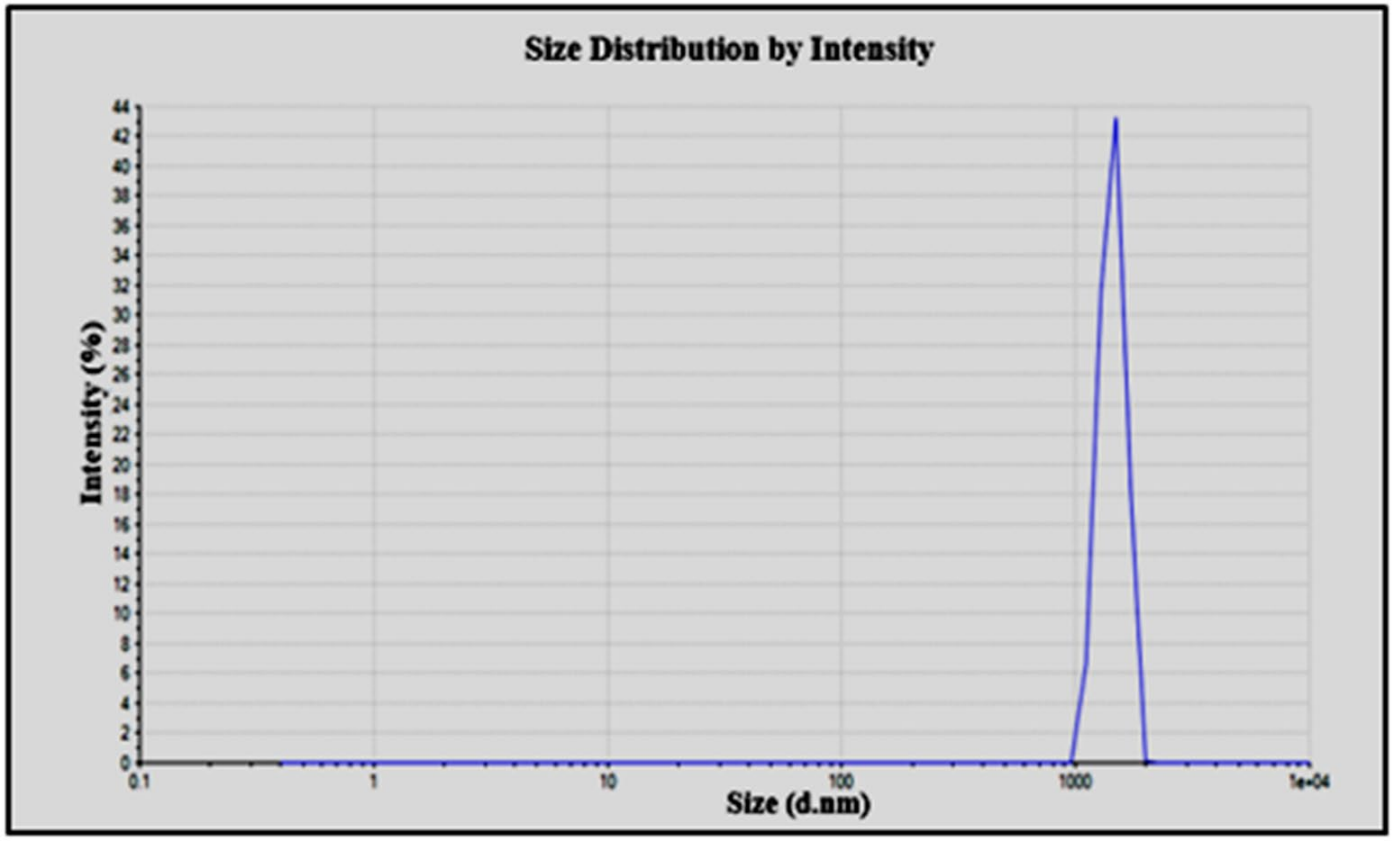
Size distribution by Intensity of FeNPs synthesized from *Phoenix dactylifera* leaves extract.

Nano Zeta-sizer using DLS strategy was used for determining the size of green synthesized FeNPs and a graph was obtained as shown in Fig. 4. This graph shows the intensity of scattering light on the y-axis and size (diameter in nanometer) on the x-axis. The size of the particle was obtained in the range of 950 to 3000 d.nm with one maximum and a sharp peak at 1438 d.nm/100.0%. Z-average size of FeNPs was 6092 d.nm with PDI of 0.909 and PDI width of 5809 d.nm.

**Figure 5:**
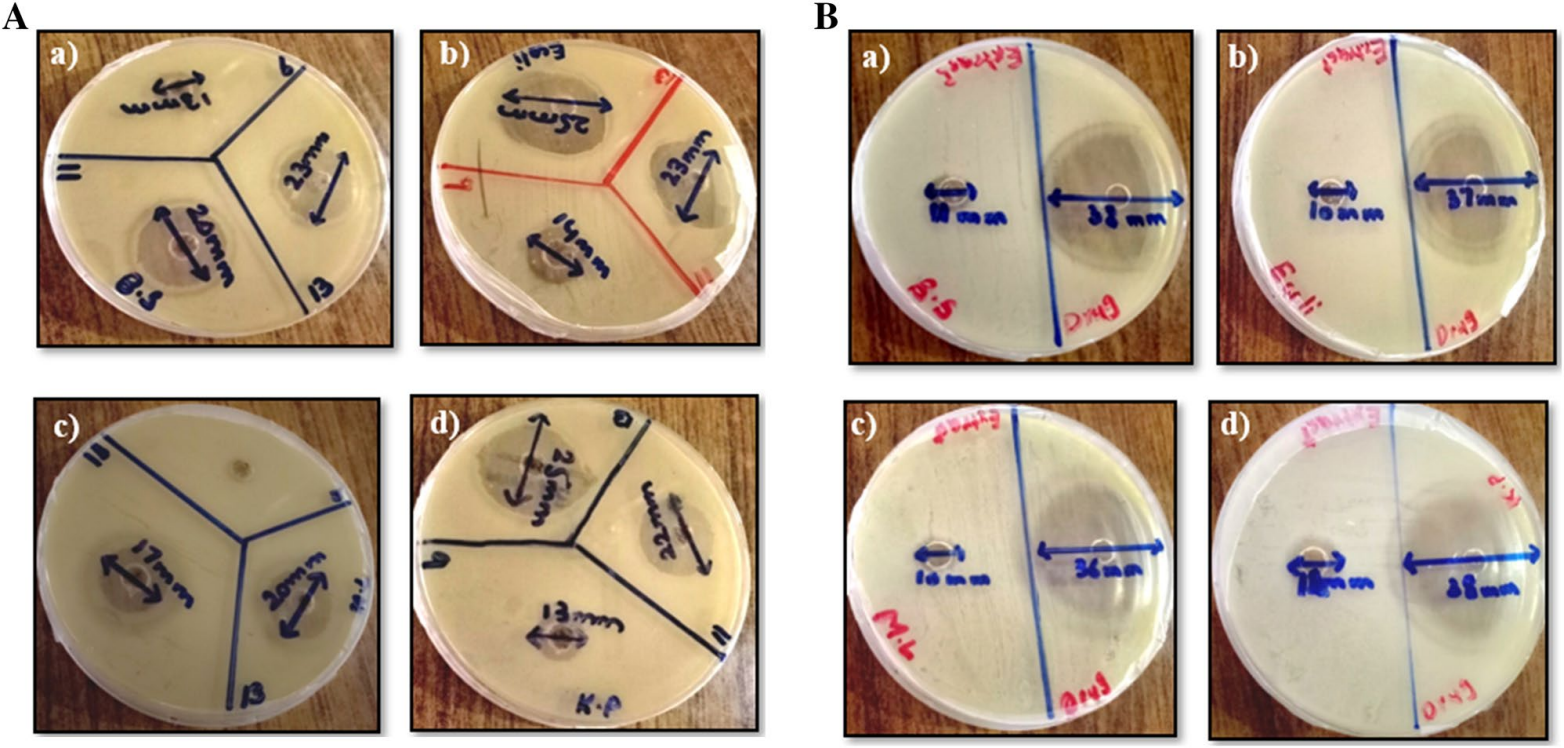
(**A)** Antimicrobial activity of synthesized FeNPs against (a) *Bacillus subtilis*, (b) *Escherichia coli*, (c) *Micrococcus leutu*s, (d) *Klebsiella pneumonia*. (**B**) Antimicrobial activity of standard drug and extract of *Phoenix dactylifera* leaves against (a) *Bacillus subtilis*, (b) *Escherichia coli*, (c) *Micrococcus leutu*s, (d) *Klebsiella pneumonia.*

The antimicrobial activity of FeNPs was evaluated by the agar well diffusion method. Zone of inhibitions for FeNPs produced by three different salt concentrations are given in Table 1 and Fig. 5A, B (Same conditions were maintained for each experiment). FeNPs synthesized by using 13 mM salt concentration showed a maximum zone of inhibition of 25 mm against *Klebsiella pneumonia* and *Escherichia coli*. While the minimum zone of inhibition i.e., 13 mm was observed for FeNPs (synthesized from 9 mM salt concentration) against *Bacillus subtilis* and *Klebsiella pneumonia.*

**Table 1:** Zone of Inhibition of FeNPs synthesized by *Phoenix dactylifera.*

Standard drug and Sample	Number of Replicates	*Bacillus subtilis*	*Escherichia coli*	*Micrococcus leutus*	*Klebsiella pneumonia*
Standard drug (ciprofloxacin)	1	37.8	36.7	35.2	37.9
	2	38.2	36.4	36.6	37.8
	3	38	38	36.2	38.3
	Mean ± SD	38 ± 0.200	37 ± 0.850	36 ± 0.721	38 ± 0.264
Aqueous extract of *Phoenix dactylifera* leaves	1	12.0	10.0	10.0	13.2
	2	11.0	10.0	10.0	10.8
	3	10.0	10.0	10.0	12.0
	Mean ± SD	11 ± 0.102	10 ± 0.250	10 ± 0.312	12 ± 0.140
9 mM FeNPs	1	12.8	13.8	0	13.4
	2	13.4	14.2	0	12.5
	3	12.8	14	0	13.1
	Mean ± SD	13 ± 0.346	14 ± 0.200	0 ± 0	13 ± 0.458
11 mM FeNPs	1	19.6	23.2	16.4	21.6
	2	19.3	22.5	17.7	22.3
	3	21.1	23.3	16.9	22.1
	Mean ± SD	20 ± 0.964	23 ± 0.435	17 ± 0.655	22 ± 0.360
13 mM FeNPs	1	22.7	24.9	18.8	25.7
	2	23.2	25.4	19.4	24.8
	3	23.1	24.7	21.8	24.8
	Mean ± SD	23 ± 0.264	25 ± 0.360*	20 ± 1.587	25 ± 0.519*

*p value < 0.05 indicates significant results compared to standard.

## Discussion

Currently, nanotechnology is a growing field of science with the most vibrant and conspicuous applications. Several types of synthesis methods of metal nanoparticles are now mentioned in literature and practiced by researchers as well to find out new and emerging applications of nanoparticles. There are three methods used for synthesizing FeNPs; physical, chemical, and biological. The biological method involves the synthesis of NPs by living organisms specifically plants, bacteria, fungi, and algae. Synthesis of NPs by plants refers to green nanotechnology^5^.

Metallic nanoparticle synthesis by using plant biomaterials is now considered an area with extensive research^17,18^. Plants are naturally composed of organic reducing agents, making them more suitable and adaptive for nanoparticle synthesis^19^. The green synthesis approach produces nanoparticles without the elimination of environmental pollutants, making it a more viable and economically efficient technology^19^. Plants are capable of reducing metallic ions on their surface as well as different tissues. Plants with a higher capacity of reduction and accumulation of metal ions are used for metal-based nanoparticle synthesis. In the green synthesis approach, the phytocompounds present in the extract of the plant are involved in the reduction of the metal ions, they also act as stabilizers of the iron nanoparticles^20^. Polyphenols, terpenoids, and flavonoids in the plant act as both a reducing agent and a capping agent resulting in the production of nanoparticles i.e. zero-valent FeNPs^21,22^.

Several plant extracts have been given a successful trial for the synthesis of metallic nanoparticles^19^. *Camellia sinensis, Azadirachta indica, Tridax procumbens, Passiflora tripartitavar, Syzygium cumini, Terminalia chebula, Salvia officinalis, Dodonaea viscose, Oolong tea,* and *Rumex acetosa* extracts are reported to synthesize different types of FeNPs with different but specific applications^23–25^. *Phoenix dactylifera* is an important plant with several medicinal applications. *Phoenix dactylifera* has been previously used for the synthesis of several other metallic nanoparticles. Such as Muhammad et al.^26^ synthesized silver nanoparticles by using leave extract of *Phoenix dactylifera* and Barani et al.^27^ also used leave extract of *Phoenix dactylifera* to synthesize zinc oxide nanoparticles. Thus, in this study, *Phoenix dactylifera* was used as a source to produce FeNPs.

Green synthesis of FeNPs was confirmed by UV–Visible spectroscopy. According to one study, the UV spectrum of FeNPs synthesized by bulb extract of *Murraya koenigii* exhibited broad absorption peaks between 275 and 500 nm^28^. Similarly, FeNPs synthesized by leaf extract of *Glycosmis mauritiana*, absorption spectra were observed between 202 and 410 nm^3^. FeNPs synthesized by *Spinacia oleracea* also exhibited absorption peaks between the range of 400–450 nm region. Flower extract mediated FeNPs of *Musa ornata* give absorption peaks between 250 and 350 nm^29^. *Tridax procumbent* mediated synthesis of FeNPs when subjected to UV analysis, displayed the highest absorption peak at 450 nm^18^. These studies are in accordance with our results as *Phoenix dactylifera* mediated FeNPs showed an absorbance peak at 450 nm.

Characterizing plant-mediated nanoparticles by FTIR spectroscopy explains the association of phytochemical components of extracts with the nanoparticle. According to the literature, terpenoids are mostly linked with nanoparticle synthesis. Terpenoids are plant-based and organic polymer which shows strong antioxidant activity. According to one study, FTIR spectra for FeNPs synthesized by leaf extract of *Platanus orientalis* were obtained by scanning the FeNP sample between the range of 400–4500 cm^-1^. Its spectrum displays stretching of the C–H group at 2096 cm^-1^ and bending of H–C–H at 1315 and 1410 cm^-1^. C–O and C–C stretching was observed at a range of about 1000–1450 (cm^−1^)^9^. Another example includes FTIR analysis of FeNPs synthesized by flower extract of *Musa ornata.* FTIR analysis was performed between the range of 400–4000 cm^−1^. Three sharp peaks at 480.69, 3383.42, and 1634.15 cm^-1^ were displayed on FTIR spectra of previously mentioned example^29^. These studies supported our results and revealed that the presence of O–H and C=O bonds due to phenol/alcohol and Alkene functional groups are involved in the synthesis and stability of FeNPs from *Phoenix dactylifera.*

FeNPs have different sizes and morphology according to their types. According to one research study, SEM analysis of iron oxide nanoparticles exhibited the size of FeNPs in the range of 58–79 nm, while the morphology of FeNPs was spherical^30^. Another example includes SEM analysis of FeNPs with a diameter of 7.7 nm synthesized by using *Passiflora foetida* extract^31^.

Jamzad and colleagues performed an experiment where they synthesized iron oxide nanoparticles using the biogenic synthesis method. FeNP nanoparticles were synthesized using the aqueous extract of the plant Laurus nobilis. The initial characterization was done through UV–Vis spectroscopy. The synthesized nanoparticles showed the maximum absorption at the range of 285 nm which indicated the synthesis of FeNPs. The FTIR spectroscopy revealed several peaks of the synthesized nanoparticle sample which indicated the functional groups which were involved in the synthesis of nanoparticles and their stability. X-ray diffraction analysis confirmed that the synthesized nanoparticles were crystalline. These iron oxide nanoparticles have notable antimicrobial activity against gram-positive bacteria^32^.

In another research conducted by Laouini et. al., synthesis of silver and silver oxide nanoparticles was done using *Phoenix dactylifera* leaves extract. Various properties of the synthesized silver nanoparticles were studied. Dye degradation property and catalytic activity of the silver and the silver oxide nanoparticles were also studied in this project. UV–visible spectroscopy (UV spectroscopy), Fourier transform infrared spectroscopy (FTIR), X-ray diffraction study (XRD), and scanning electron microscopy (SEM) were done. The UV spectroscopy showed that the silver nanoparticles had absorption peaks at 430 nm which is the reported wavelength range for silver nanoparticles. The spherical shape of the silver nanoparticles was confirmed by the SEM and various functional groups which were involved in the synthesis and stability of the nanoparticles were confirmed by FTIR spectroscopy. The silver nanoparticles were crystalline and their size was about 28.66–39.40 nm and it was confirmed by XRD. This study reported that the synthesized nanoparticles had notable catalytic activity against the degradation of the dyes^33^.

According to numerous research-based studies, FeNPs have evident antimicrobial activities against bacterial cultures including *Escherichia coli, Staphylococcus aureus, Salmonella enteric, Pseudomonas aeruginosa, Streptococcus pyogenes, Aeromonas hydrophila, Klebsiella pneumonia, Bacillus cereus,* and *Enterococcus faecalis*^17,34^*.* In a study, researchers evaluated the antibacterial activity of FeNPs against *Escherichia coli*, *Pseudomonas aeruginosa,* and Staphylococcus *aureus* by using different ratios of FeSO_4_ salt and *Azardirachta Indica* leaf extract^35^. Another study reported the antibacterial activity of iron oxide nanoparticles synthesized from *Punica granatum* peel extract. Results of this study reported that a maximum zone of inhibition (22 ± 0.5) was observed against *Pseudomonas aeruginosa *^36^. Our results are also in accordance with these studies. These findings are consistent with the proposed mechanism of antimicrobial action of FeNPs involving particle accumulation in the cytosol. The smaller the nanoparticle, the greater the penetration and accumulation capacity of nanoparticles within the bacterial cell wall. Nanoparticles should cause the rupture of the bacterial cell membrane by which cellular content fugues take place^37,38^. Moreover, small-sized nanoparticles have a larger surface area which causes the conformational changes in bacterial cell DNA and as a result, causes bacterial cell death. This could also be a mechanism involved in the antibacterial potential of FeNPs^39^. It might explain why FeNPs have the different antibacterial potential for different types of bacteria; because gram + ive bacteria have a thick peptidoglycan membrane, there is likely a high degree of contact between organisms and nanoparticles due to their small size. Instead of a cytoplasmic membrane, gram -ive bacteria have a cytoplasmic membrane and an outer cell membrane, with only a thin film of peptidoglycan between them. It is extremely difficult for FeNPs to penetrate the thin layer in this situation^40^.

Another hypothesized mechanism for the antibacterial activity of FeNPs is the generation of reactive oxygen species (ROS), like hydroxyl radicals and singlet oxygen inside the bacterial cell. The phenomenon of ROS occurs due to the Fenton reaction of Fe and metabolic products e.g. hydrogen peroxide of bacterial cells^35,41^. ROS produce oxidative stress in bacteria cells, resulting in bacterial mortality. Though the antibacterial mechanism of action of FeNPs is not very clear, however, it is evident that these nanoparticles could act as potential antibacterial agents.

Along with prominent antimicrobial activity, FeNPs also have numerous other applications in different fields including medical, catalysis, environmental, and magnetic areas^9,42,43^.

## Conclusion

FeNPs were successfully synthesized by a green method using *Phoenix dactylifera* leaf extract. Synthesis of FeNPs was confirmed by a notable color change of the sample from yellow to black and initially characterized by UV Visible Spectrophotometer with a sharp peak at 450 nm. Further characterization of green synthesized FeNPs was done through FTIR and nano Zeta-sizer for detection of the responsible functional group of phytochemical constituents of the *Phoenix dactylifera* extract and size of synthesized FeNPs respectively. FTIR absorbance bands of FeNPs were observed at 1636.34 and 3282.19 cm^−1^. The presence of the O–H (phenol or alcohol) functional group was confirmed by the absorbance band at 3282.19 cm^-1^ and the Alkene group present in the wave region 1636.34 cm^−1^ was also confirmed. The size of the particle using Nano Zeta-sizer was obtained in the range of 950–3000 d.nm with one maximum and sharp peak at 1438 d. nm/100.0%. Z-average size of FeNPs was 6092 d.nm with PDI of 0.909 and PDI width of 5809 d.nm. Antimicrobial activity pf FeNPs was performed against four bacterial strains including *Klebsiella pneumonia, Bacillus subtilis, Micrococcus leutus,* and *Escherichia coli.* Maximum antimicrobial activity was observed for FeNPs (synthesized from 13 mM salt concentration) against *Escherichia coli* (25 ± 0.360) and *Klebsiella pneumonia* (25 ± 0.519). Aqueous extract of *Phoenix dactylifera* offers an eco-friendly and cost-effective method to synthesize FeNPs which could be pave way for diverse applications, especially as antimicrobial agents.

## Materials and methods

### Collection of materials

Experimental research procedures on plants were accomplished according to the institutional guidelines of the Institute of Molecular Biology and Biotechnology, Bahauddin Zakariya University, Multan, Pakistan. Fresh leaves of *Phoenix dactylifera* were collected from Biopark, Bahauddin Zakariya University, Multan, Pakistan in 2019. The plant parts were cleaned with tap water and were identified by Dr. Zafar Ullah Zaffar, Taxonomist, Department of Botany, Bahauddin Zakariya University, Multan, and the specimen was deposited in the herbarium of the institute with voucher No. IMBB 1923.

Iron sulfate heptahydrate (Fe_2_SO_4_·7H_2_O) was purchased from DaeJung Chemicals, Lahore, Pakistan.

### *Phoenix dactylifera* extract preparation

20 g fresh date palm tree leaves were washed thoroughly with distilled water to remove dirt particles and then dried in the laboratory for 20 min^44^. Leaves were chopped into small pieces by scissors and boiled in 100 ml distilled water^45^. Boiling of the extract was done on a water bath for 30 min at 70 °C^38^. The extract was cooled at room temperature and then filtered using 125 mm filter paper. The color of the extract was dark yellow^1^.

### Preparation of salt solution

1 molar stock solution of Fe_2_SO_4_.7H_2_O was prepared in 50 ml of distilled water. 9 mM, 11 mM, and 13 mM dilutions of stock solution were prepared^46^.

### Synthesis of iron nanoparticles (FeNPs)

Using the green synthesis method of nanoparticles, *Phoenix dactylifera* leaves extract was used for the reduction and capping of Fe ions^47^. 1:10 sample solutions were prepared by adding 2 ml of extract in 20 ml of each 9, 11, and 13 mM Fe_2_SO_4_·7H_2_O solution at normal room temperature in a 100 ml Erlenmeyer flask. For the preparation of different concentrations of iron salt solutions, a stock solution was prepared in 50 ml of distilled water^48^. The solutions were continuously stirred for 8 h at 60–70 °C and then subjected to further stirring at 37 °C for 24 h^29,49^.

### Characterization of FeNPs

Characterization of synthesized FeNPs was conducted through the following techniques.

#### UV Visible Spectrophotometry

After a visible color change of the sample solutions, further confirmation of synthesis of FeNPs was done through UV Visible Spectrophotometer^50^. 1 ml of nanoparticle sample of each concentration (9 mM, 11 mM, and 13 mM) was used for analysis. UV analysis of these samples was conducted after 24 h of incubation. Absorbance was measured through UV visible spectrophotometer at 1 nm resolution^51,52^. The spectrophotometer used for measurement of the wavelength of synthesized FeNPs was PG instrument-t80 UV/VIS Spectrophotometer.

#### Fourier transform infrared spectroscopy (FTIR)

FTIR is used for scanning the sample and it gives information about the relevant functional groups and stabilization of synthesized FeNPs^53–55^. A detector can present the resulting spectrum in the range between 4000 and 400 cm^−1^.

For FTIR analysis, the FeNP samples were freeze-dried into powder form and diluted in Potassium Bromide with 1:100. The resulting spectrum was obtained in the range of 1000 to 3500 cm^-1^ wavelength^56^. FTIR Spectroscopy was carried out by using Bruker Germany Alpha FTIR spectrophotometer.

#### Dynamic light scattering (DLS)

The Zeta-sizer nano series can carry outsize measurements by using a phenomenon called Dynamic Light Scattering (DLS). The Zeta-sizer nano range of instruments can help to evaluate three significant characteristics of particles within a liquid medium. These three fundamental parameters include particle size, molecular weight, and zeta potential^57,58^. For operating Zeta-sizer, a sample amount of minimum of 12 µL and a maximum of 3 ml and temperature range of 15–40 °C is required^46,59^. This procedure was carried out by using Zetasizer Nano S90.

### Evaluation of anti-microbial activity

The antimicrobial activity of synthesized nanoparticles was assessed against four bacterial cultures including *Escherichia coli*, *Bacillus subtilis*, *Micrococcus leutus,* and *Klebsiella pneumonia* by using the agar well diffusion method. All of the mentioned bacterial cultures were locally isolated, identified, and maintained by the Institute of Molecular Biology and Biotechnology, Bahauddin Zakariya University, Multan, Pakistan. For inoculum preparation, the nutrient broth was prepared and bacterial cultures were mixed with the help of a sterilized wire loop in nutrient broth and subjected to incubation at 37 °C for 24 h. For the agar, well diffusion method, a metallic borer with 6 mm size was used for making wells and ciprofloxacin was used as a standard drug.

### Statistical analysis

The true experimental research design was used for performing the experiments. SPSS ver. 22 was used for analyzing the data. p < 0.05 was considered significant.

### Experimental research procedures on plants

Experimental research procedures on plants were accomplished according to the institutional guidelines of the Institute of Molecular Biology and Biotechnology, Bahauddin Zakariya University, Multan, Pakistan.

### Ethical approval

Experimental organisms were not used.

## Data Availability

All the other data that support the findings of this study are available from the corresponding author upon request.
